# How wasting is saving: Weight loss at altitude might result from an evolutionary adaptation

**DOI:** 10.1002/bies.201400042

**Published:** 2014-06-11

**Authors:** Andrew J Murray, Hugh E Montgomery

**Affiliations:** 1) Department of Physiology, Development & Neuroscience, University of CambridgeCambridge, UK; 2) UCL Institute for Human Health and PerformanceLondon, UK

**Keywords:** amino acids, catabolism, hypoxia, ketones, metabolism, muscle

## Abstract

At extreme altitude (>5,000 – 5,500 m), sustained hypoxia threatens human function and survival, and is associated with marked involuntary weight loss (cachexia). This seems to be a coordinated response: appetite and protein synthesis are suppressed, and muscle catabolism promoted. We hypothesise that, rather than simply being pathophysiological dysregulation, this cachexia is protective. Ketone bodies, synthesised during relative starvation, protect tissues such as the brain from reduced oxygen availability by mechanisms including the reduced generation of reactive oxygen species, improved mitochondrial efficiency and activation of the ATP-sensitive potassium (K_ATP_) channel. Amino acids released from skeletal muscle also protect cells from hypoxia, and may interact synergistically with ketones to offer added protection. We thus propose that weight loss in hypoxia is an adaptive response: the amino acids and ketone bodies made available act not only as metabolic substrates, but as metabolic modulators, protecting cells from the hypoxic challenge.

## Introduction

Human acclimatization to the sustained reduction in cellular oxygen availability of high altitude (hypobaric hypoxia) relies not just on mechanisms to sustain oxygen *delivery* to the tissues [1], but on alterations to oxygen *use* [2, 3]. Both mechanisms are regulated, at least in part, by the hypoxia inducible factor (HIF) family of transcription factors, which drive the expression of (thus) ‘hypoxia-sensitive’ genes [4].

### Weight loss at altitude results partly from decreased energy intake

Hypoxia (whether normobaric or hypobaric) is associated with significant involuntary fat and muscle loss [5, 6] to which a variety of factors, including exertional metabolic demands and elevated basal metabolic rate, may contribute [7–9]. However, whilst altitude exposure increases perceived exercise intensity, actual energy expenditure is much lower, with oxygen consumption rates falling as altitude increases [10]. Meanwhile, malabsorption of nutrients and a reduction in appetite – perhaps partly mediated through increased circulating levels of the satiety hormones cholecystokinin (CCK) [11] and leptin [12] – reduce energy intake by as much as half [5, 13]. Decreased energy intake thus appears to be the dominant cause of weight loss at altitude (albeit that overall energy expenditure might rise as total exertional load increases), particularly since any increase in activity does not seem to elicit a corresponding increase in energy intake [10] and since diet-induced thermogenesis also falls along with intake [9].

### At higher altitudes, loss of muscle mass is greater than fat loss

At higher altitudes (above 5,000–5,500 m), loss of muscle mass accounts for a greater proportion (66–73%) of overall weight loss than does fat loss [5, 6], perhaps as a result of additional, evolutionarily ubiquitous, direct effects of hypoxia on protein synthesis [14]. These effects are mediated through inhibition of gene transcription and translation [15] and by induction of the metabolic sensor, AMP kinase (AMPK), which inhibits protein synthesis via the target-of-rapamycin kinase (mTOR) pathway whilst promoting muscle catabolism [16].

### The consequences of inadequate food intake and of muscle breakdown

With inadequate food intake, lipolysis of triacylglycerol stores releases sufficient fatty acids that the quantities of acetyl-CoA so generated can overwhelm the Krebs cycle. In this instance, acetyl-CoA is diverted towards hepatic ketone biosynthesis, yielding acetoacetate (AcAc) and β-hydroxybutyrate (β-OHB). In the mitochondria of distant tissues, such as brain and heart, the ketone bodies are reconverted to acetyl-CoA for entry into the Krebs cycle.

**Figure 1:**
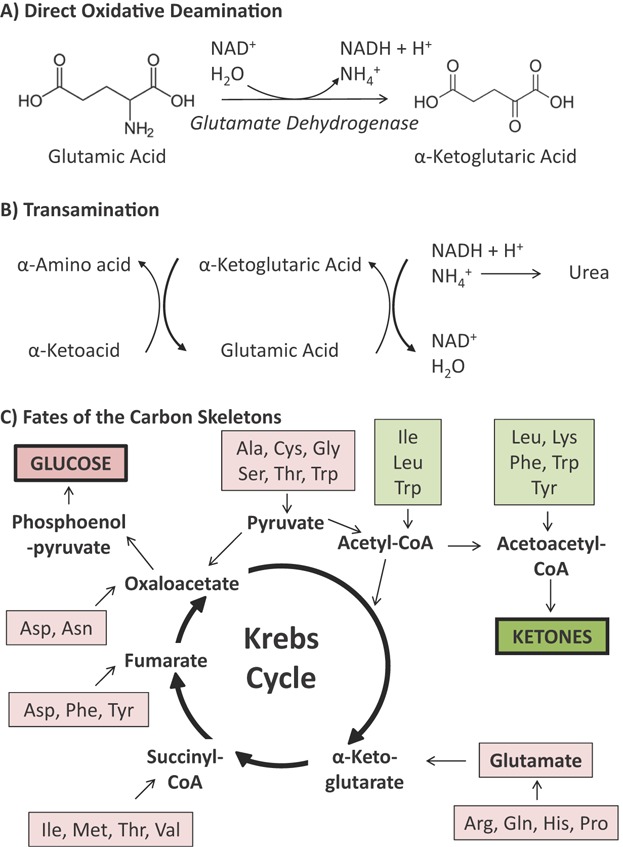
Pathways of amino acid breakdown. **A:** Some amino acids (e.g. glutamic acid/glutamate) can undergo direct deamination to their corresponding keto-acids (e.g. α-ketoglutaric acid/α-ketoglutarate). **B:** Most amino acids undergo initial transamination, with the amino group transferred to α-ketoglutarate, to yield a ketoacid and glutamate, which can then undergo direct deamination as above. **C:** The ketoacids resulting from the above reactions are converted to a small number of metabolites that fuel the Krebs cycle. As such, some amino acids are glucogenic (pink boxes), yielding glucose, whilst others are ketogenic (green boxes) and yield acetoacetyl-CoA, and thus, ketone bodies. Note that some amino acids can be either glucogenic or ketogenic. Ala, alanine; Arg – arginine; Asn, asparagine; Asp, aspartate; Cys, cysteine; Gly, glycine; Gln, glutamine; His, histidine; Ile, isoleucine; Leu, leucine; Lys, lysine; Met, methionine; Phe, phenylalanine; Pro, proline; Ser, serine; Thr, threonine; Trp, tryptophan; Tyr, tyrosine; Val, valine.

Meanwhile, protein breakdown releases amino acids. Some amino acids can undergo direct oxidative deamination to their corresponding ketoacids in the liver (e.g. glutamate is converted to α-ketoglutarate by glutamate dehydrogenase, whilst glycine is converted to glyoxylate by glycine oxidase), yielding reduced NAD (NADH) and a free ammonium ion (Fig. 1A), which is converted to urea and excreted. Most amino acids, however, are initially transaminated rather than deaminated, the amino group being transferred to α-ketoglutarate to yield a ketoacid and glutamate, which can itself then undergo direct deamination (Fig. 1B). The resulting ketoacids – essentially the carbon skeletons of the deaminated amino acids – enter pathways that converge on just a handful of molecules that between them fuel the Krebs cycle (Fig. 1C). Alanine, serine, cysteine, tryptophan and glycine, for instance, can yield pyruvate; glutamine, proline, arginine and histidine are converted to glutamate and thence to α-ketoglutarate; whilst succinyl-CoA, fumarate and oxaloacetate can arise from other amino acids. Amino acids that are degraded to these Krebs cycle intermediates are termed glucogenic amino acids. Meanwhile, the so-called *ketogenic* amino acids are converted to acetoacetyl-CoA or acetyl-CoA, and can give rise to ketone bodies. Whilst only leucine and lysine, of the 20 canonical amino acids, are solely ketogenic, isoleucine, phenylalanine, tryptophan and tyrosine are both ketogenic and glucogenic. Thus, protein catabolism from muscle breakdown can further drive hepatic ketone biosynthesis.

### Hypothesis

We propose that the catabolic response to altitude (comprising both lipolytic and myolytic components) is orchestrated, and that it is both metabolically advantageous and protective under hypoxic conditions.

## Advantages of the catabolic state

### Improvements in oxygen supply/demand matching

So what might be the advantages of such a hypoxia-induced cachectic state? First, of the 80% of mitochondrial oxygen consumption that is estimated to be coupled to ATP synthesis, at rest 25–30% is accounted for by protein synthesis [17]. A fall in protein synthesis would thus be advantageous when oxygen is scarce [15]. Indeed, in some animals ATP demand for protein synthesis can fall by 93% under hypoxic conditions [14]. Second, the ensuing weight loss might itself improve the economy of movement [18]. Meanwhile, thinner myocytes also contribute to an enhanced muscle capillary density [19, 20] and therefore perhaps improved oxygen delivery as a result of shorter diffusion distances. Finally, loss of muscle results in loss of total mitochondrial mass, whilst with prolonged exposure to hypoxia at high altitude, mitochondrial density falls in the remaining muscle, further decreasing oxygen demand [3, 21].

### Ketones as metabolic substrates

But could there be an advantage to the release of amino acids and ketone bodies? Certainly both are metabolic fuels. Ketone bodies, in particular, can substitute for glucose as energy substrates for the brain, and indeed most other tissues, during starvation and heavy exercise [22]. Concentrations of β-OHB, for instance, are normally very low (∼0.1 mmol/L), but can rise 13-fold in humans during prolonged starvation, with increased cerebral ketone uptake contributing substantially to cerebral metabolism [23]. Under conditions of starvation or exercise, when exogenously acquired glucose becomes depleted, there is a clear advantage to promoting the hepatic synthesis of ketones – largely derived from endogenous fat reserves – because fatty acids are themselves unable to cross the blood-brain-barrier, and are thus able to make only a negligible contribution towards satisfying cerebral energy demands. However, the advantage of reducing calorie intake at altitude, only to substitute exogenous energy sources with those endogenously derived, is not obvious, particularly since, in general, natural selection has favoured a state of positive energy balance in humans [24].

### Ketones lower the oxygen cost of ATP synthesis compared with fatty acids

It is likely, therefore, that ketones offer some advantage over glucose as a fuel. But what might that be? Certainly, the elevation in β-OHB seen in prolonged human starvation is associated with a decrease in respiratory quotient (RQ; CO_2_ produced/O_2_ consumed) [25]. Whilst the ventilatory cost associated with CO_2_ clearance would therefore be reduced under such circumstances, it is doubtful that this alone would offer significant advantage to survival or performance at altitude, particularly since, in the context of altitude, ventilatory drive is increased because of hypoxia [1]. The ATP yields of ketone and glucose oxidation per 2-carbon unit are similar, and in this regard both are inferior to fatty acids [22]. Whilst, during starvation, lipolysis of triglyceride stores might enhance fatty acid availability and drive oxidation in tissues other than the brain, the increased ATP yield comes at a price. The oxygen cost of fatty acid oxidation is greater than that of either ketones or glucose, and is worsened further by the fatty acid driven activation of mitochondrial uncoupling proteins (UCPs) and peroxisomal fatty acid oxidation, which yields no ATP [22]. Indeed, under sustained hypoxic conditions, fatty acid oxidation is suppressed in cultured cells and human skeletal muscle [3, 21].

So is this apparent switch to endogenous fuels simply the price of catabolism? Or could there be advantages to increased ketone and amino acid flux?

## The advantages of enhanced ketone synthesis

That a switch to ‘different fuels’ might be advantageous is suggested by the fact that hypoxia not only drives ketogenesis through relative starvation, but also directly *augments* ketone synthesis, just as it does protein catabolism: in neurons, at least, hypoxia induces AMPK, which itself may enhance ketone flux [26]. Also suggestive of an advantage to ketogenesis in these circumstances is the fact that ketones protect the brain in situations where the balance of oxygen/substrate delivery and use is unfavourable, for example during ischaemia/hypoxia; β-OHB reduces cerebral infarct size following ischaemic stroke [27, 28].

### Ketones decrease reactive oxygen species production via uncoupling

Such effects may be partly mediated via decreased reactive oxygen species (ROS) production [29]. Ketogenic diets raise hippocampal glutathione peroxidase activity fourfold [30] and mitochondrial uncoupling protein 2 (UCP2) expression by 55%, reducing ROS production to a similar degree [31]. Likewise, consumption of a novel ketone ester diet increased levels of the other brain uncoupling protein isoforms, UCP4 and UCP5, to a similar extent [32]. Although increased uncoupling might exacerbate tissue hypoxia, due to the increased oxygen required in order to maintain ATP synthesis, the antioxidant effect elicited by mild uncoupling might, under these circumstances, outweigh the harm.

### Ketones protect mitochondrial function independently of effects on ROS production

Ketones seem to have other protective mitochondrial effects, however, independent of those mediated through ROS modulation. Rotenone (an inhibitor of the mitochondrial electron transport chain complex I) causes dose-dependent synaptic inhibition independent of changes in ROS levels (and unaffected by the application of antioxidants), whilst ketone supplementation reverses the rotenone-induced decrease in ATP levels [33]. Ketones divert glucose from oxidative metabolism towards replenishment of Krebs cycle intermediates (*anaplerosis*), one of which (propionate, metabolized to succinyl-CoA to enter the Krebs cycle), protects the heart from ischaemia-reperfusion injury [34]. Meanwhile, the cytotoxic agent 1-methyl-4-phenylpyridinium inhibits cultured neuronal mitochondrial NADH dehydrogenase activity, impeding electron transport and increasing free radical production, leading to ATP depletion and cell death. Exposure to β-OHB increases cell survival under such circumstances [35], as it does when such cells are exposed to rotenone [36].

### Ketones improve mitochondrial metabolic efficiency

Ketone exposure also improves mitochondrial metabolic efficiency: oxygen consumption in isolated perfused working rat hearts falls, whilst mechanical work output increases [37]. Similar effects are seen in mammalian sperm, whose motility is increased with β-OHB exposure, whilst oxygen consumption falls [38]. Such effects may be mediated, in part, through oxidation of the coenzyme Q couple and reduction of the nicotinimide adenine dinucleotide (NAD) couple; this increases energy release as an electron travels the electron transport chain, and thus the free energy release from ATP hydrolysis. In essence, the reactants in one step of ATP synthesis (NADH/NAD^+^), become more reduced while those at the next step (Coenzyme Q/Coenzyme QH_2_), become more oxidized, ‘widening the energetic gap between the two’ [39]. Such a gain in metabolic efficiency would clearly be advantageous under conditions where oxygen availability is relatively low.

### Ketones elicit hypoxic protection via effects on K_ATP_ channels

Ketones may also regulate metabolism (and offer hypoxic protection) through effects on ATP-sensitive potassium channels (K_ATP_ channels). These have two integral subunits: the inward rectifier K channel 6 (Kir6.1/Kir6.2, a pore-forming subunit) and the sulfonylurea receptor (SUR), inhibited by ATP and sulfonylurea drugs, respectively. Conversely, nucleotide hydrolysis by SUR causes channel opening, maintained by the resultant Mg-ADP. The ATP/ADP ratio is thus a key determinant of K_ATP_ state. K_ATP_ channels thus function as molecular rheostats, matching cellular energy demands to membrane potential-dependent functions. Short periods of ischaemia preserve high-energy phosphate levels and cellular viability in the face of more severe and prolonged ischaemia (‘ischaemic preconditioning’), a mechanism in which mitochondrial K_ATP_ channels are believed to play a crucial role [40]. Although ischaemia and hypoxia are not the same stress, common elements (including, but not confined to, HIF-1) mediate both hypoxic adaptation and ischaemic protection [41, 42], with chronic hypoxia increasing SUR2A protein (and thus K_ATP_ channel) expression and protecting cells from metabolic stress [43]. Meanwhile, the aryl hydrocarbon receptor nuclear translocator (ARNT) – which binds the aryl hydrocarbon receptor and HIF-1/HIF-2α – regulates the response to hypoxia, but also regulates expression of the Kir6.2 subunit, and thus of the K_ATP_ channel itself [44]. Of relevance to our hypothesis, ketones may open the K_ATP_ channel, eliciting the neuronal effects that might underlie their anticonvulsant properties [45]. There is, of course, no reason to believe that such effects would be confined only to neuronal channels. Indeed, ketones also open K_ATP_ channels in myocytes [46]. In summary, when cellular energetics are compromised by hypoxia, K_ATP_ channel opening preserves viability and function [47] – and this might be augmented by ketones.

### Ketones interact with HIF signalling pathways

Thus, hypoxia induces catabolism and the synthesis of ketones, which act as metabolic regulators. Additionally, ketones may also interact with hypoxia signalling itself. Ketone utilization elevates intracellular succinate, increasing HIF-1 levels [48]. Increased cerebral HIF-1, induced by β-OHB or a ketogenic diet, is associated with reductions of 55–70% in cerebral infarct volumes in experimental ischaemic stroke [49]. Thus, ketones may both mediate HIF-dependent responses to hypoxia, and also help to regulate them. In the context of hypoxia, ketones help maintain mitochondrial membrane potential and decrease hippocampal neuronal death [50], and prevent rises in rat cerebral lactate concentrations [51]. For all of these reasons, ketones might benefit all aerobic cells exposed to hypoxia [39].

## The advantages of enhanced amino acid availability

### Inhibition of protein synthesis and regulation of mitochondrial function by mTOR

Whilst hypoxia inhibits mTOR, and thus protein synthesis [52, 53], mTOR is also a key regulator of mitochondrial function and oxidative capacity [54]. It thus orchestrates both a catabolic and metabolic response to hypoxia. The branched chain amino acids interact with mTOR to modulate this response [55] whilst having their own direct effects: leucine, for instance, inhibits mitochondrial oxidative phosphorylation, thereby decreasing oxygen demand [56]. Amino acid release from muscle might maintain availability (and thus hypoxic protection) when ingestion is reduced in order to drive ketone synthesis. Alternatively, the catabolic response might augment cellular availability beyond that found under normal, fed, conditions, when insulin stimulates the uptake of amino acids into tissues and the synthesis of proteins in a variety of ways. In addition to possible roles as metabolic substrates in their own right, a number of amino acids elicit protective responses that might be beneficial under hypoxic conditions.

### Glycine protects renal tubules in hypoxia

Glycine protects isolated renal proximal tubule cells against a wide range of chemical inhibitors of oxidative phosphorylation (e.g. rotenone, cyanide and carbonyl cyanide *m*-chlorophenylhydrazone (CCCP)) without itself supporting cellular respiration and ATP synthesis [57]. Moreover, glycine offers potent, ROS-independent, hypoxic protection to isolated renal proximal tubular cells [58] and human umbilical endothelial cells [59]. Such effects seem to be highly structurally specific, being confined (in studies of more than 45 amino acids and analogues) to glycine and beta-, l- and d-alanine [60]. Moreover, the effects seem to be independent of amino acid metabolism [60, 61], glutathione accumulation [57] or changes in intracellular pH [62], and currently remain inadequately explained [63]. Whatever the mechanism, these amino acids may play an important role in the protection of (at least) renal tubular cells in the face of hypoxia.

### Taurine protects mitochondrial function

Meanwhile – taurine, not one of the canonical 20 amino acids encoded by DNA but one that is derived from cysteine – protects against ischaemia-reperfusion injury [64, 65] and improves respiratory chain activity in sepsis [66]. Regardless of whether taurine exerts these effects solely through its antioxidant properties or through non-mitochondrial effects (including altered ROS activity), effects on mitochondrial function, including protection against arsenic-induced apoptosis and oxidative stress [67] and improved calcium buffering [68], *have* been demonstrated.

### Glutamine regulates the cellular response to hypoxia

Plasma glutamine is a mitochondrial substrate, and a glutamate precursor. Hypoxia stimulates its uptake and regulates its metabolism in a variety of cells [69–72]. Consistent with such increased flux, plasma levels fall in humans exposed to high-altitude for three weeks [73], despite presumed increased release due to skeletal muscle catabolism over this time. However, glutamine is not just a substrate, but a metabolic modulator [74] regulating the response to hypoxia. It protects cells from oxidative stress [75], preserves anoxic heart function [76], protects the gut [77], lung [78], liver [79] and kidney [80] from ischaemia/reperfusion injury, modulates the cardiac preconditioning response [81] and acts as a preconditioning agent in its own right [82]. Such effects may, to some degree, be HIF-dependent: in human prostate (DU-145) and pancreatic (MiaPaCa-2) cancer cells exposed to hypoxia, deficiency of glutamine (but not pyruvate) suppresses the associated rise in HIF-1α levels, seemingly through translational disruption [83]. Glutamate, meanwhile, is a powerful modulator of the activity of adenosine monophosphate (AMP)-activated kinase (AMPK), a metabolic sensor [84, 85]; at least in tumours, it is essential for the expression of hypoxia-induced factors such as vascular endothelial growth factor [86].

## Synergistic effects of elevated ketones and amino acids

Thus, both ketones and amino acids may act independently as both metabolic fuels and metabolic modulators. In addition, however, amino acids released from the skeletal muscle pool might also act *synergistically* with ketoacids to further regulate the human metabolic response to hypoxia. Thus, glutamic acid can be deaminated by glutamate dehydrogenase to yield α-ketoglutarate. Supplementation of α-ketoglutarate (α-KG) in combination with aspartate both prevents and reverses hypoxia/reoxygenation-induced impairments in mitochondrial metabolism (and of complex I function, specifically) [87]. Anaerobic metabolism of these agents yields ATP and maintains mitochondrial membrane potential, as do other citric acid cycle intermediates that can promote anaerobic metabolism, such as fumarate or malate (whether alone, or in combination with α-KG). Succinate, the end-product of these anaerobic pathways, can bypass complex I, but it protects only when applied during reoxygenation and not when applied only during hypoxia [87]. Thus, renal tubule cells subjected to hypoxia/reoxygenation suffer complex I dysfunction, which is prevented/reversed by citric acid cycle metabolites that anaerobically generate ATP and which maintain mitochondrial membrane potential via electron transport at complex I [88]. The pathways that allow metabolism of citric acid cycle intermediates exist in other human tissues, and may thus represent a means of more general systemic protection in the face of hypoxia [59, 88].

## Future studies, clinical and evolutionary perspectives

### Does the hypoxic catabolic state differ from the response to fasting alone?

The hypoxic catabolic state (which we propose to be beneficial) thus appears largely due to a negative energy balance resulting from prolonged restriction of caloric intake. However, this is augmented by hypoxic inhibition of skeletal muscle protein synthesis. In addition, unlike the usual metabolic response to fasting, hypoxic acclimatization is associated with a shift in substrate preference away from, rather than towards, fatty acid oxidation in cardiac [89] and skeletal muscle [21]. The difference may in part lie with the regulation of peroxisome proliferator-activated receptor α (PPARα) and its downstream factors. Suppressed (at least in some tissues) in a HIF-1-dependent manner in the hypoxic setting [90], PPARα is, by contrast, a vital factor in the response to normoxic starvation, with mice deficient in PPARα failing to activate pathways of fatty acid oxidation and promote hepatic ketogenesis [91]. In liver, activity of the (mTOR-containing) mTOR complex 1 (mTORC1) is suppressed in fasting, hence relieving inhibition of PPARα and thereby promoting fasting-induced ketogenesis [92]. Hypoxic inhibition of mTOR itself, which in muscle inhibits protein synthesis, might therefore rescue the PPARα-supported hepatic ketogenesis under hypoxic conditions, perhaps underlining the importance of this mechanism for survival.

### Further studies

The nature of the hypoxic catabolic state, and how it might differ from starvation or calorie restriction under normoxic conditions, thus clearly deserves further attention. Metabolic flux studies, with particular attention paid to ketone biosynthesis and oxidation, could establish the changes in substrate flux that occur in response to prolonged hypoxia. Such studies could be carried out in humans, either at altitude or in hypoxic chambers, or in animal models of hypoxia. Pair-fed normoxic control subjects would be vital components of all of these studies in order to establish the precise role of hypoxia – as a distinct contributor from the reduced calorie intake per se – in the metabolic reprogramming that occurs in these states. The effects of achieving ketosis at altitude by dietary means might also deserve investigation. Would a ketogenic diet or non-toxic ketone ester supplementation prevent muscle wasting at altitude or in simulated hypoxia, and might it improve exercise efficiency? Moreover, it would be interesting to investigate whether those individuals that lose the most weight at altitude, and particularly those who predominantly lose lean mass, fare better in terms of exercise performance. Further, are these patterns of weight loss, largely observed in lowlanders, replicated in high-altitude natives? In addition, the putative protective effects of amino acids at physiological concentrations on mitochondrial function – whether alone or in combination with ketone bodies – deserves further study in the light of recent technical developments including more sensitive means of measuring oxygen consumption and ROS generation.

### Clinical and evolutionary perspectives

Given that humans probably evolved close to sea level, the human cachectic response to high altitude, at least in lowland natives, is unlikely to have resulted from altitude-related selection pressure. Perhaps a response of value at sea level, has later shown benefit at altitude?

Thus, the reduced oxygen availability associated with haemorrhage (such as that which occurs near the time of childbirth) engenders an erythropoietic response, which helps restore oxygen transport. However, an exaggerated erythrogenic response to the hypoxia of altitude can prove lethal [93]. Indeed, later natural selection in Tibetan natives has favoured lower circulating haemoglobin levels than those seen in lowlanders ascending to altitude [94].

Perhaps the catabolic state represents an advantageous adaptive response at sea level, the benefit of which becomes clearer upon exposure to the severe systemic hypoxia of altitude? The human response to the hypoxia of high altitude shares many features with disease states in which reduced oxygen availability plays a role [95]: similar patterns of weight loss, muscle wasting and altered cellular metabolism are noted in chronic obstructive pulmonary disease (COPD) [96, 97] and heart failure [98], for instance. Perhaps wasting offers a survival advantage when disease states are complicated by reduced cellular oxygen delivery, and it is this response, which has been selected for.

Likewise, in the critically ill, loss of muscle mass occurs early and rapidly, and is associated with an inhibition of protein synthesis [99]. Loss of adipose mass also occurs. Could it be that these responses are protective rather than maladaptive? One is minded of the folklore advice to ‘feed a cold, but starve a fever’. Certainly, the administration of parenteral nutrition (when enteral nutrition is not possible) does not improve patient outcome in the critically ill [100], and active efforts to supplement intake in order to meet calorie targets may in fact be harmful [101]. Perhaps, just as at altitude, cachexia in ICU might offer short or medium-term metabolic advantages that aim to protect against cellular injury, albeit that this may ultimately lead to profound weakness in many patients [102]. The potential for ketosis as a therapeutic intervention, or the administration of ketone bodies as an energy source, thus deserves investigation, and might obviate the need for muscle degeneration in order to support survival at all costs.

Thus, whilst cachexia in response to hypoxia at altitude may offer a survival or performance advantage, it perhaps has its origins in protection under circumstances in which tissue oxygen delivery was impaired due to injury or illness, for instance following haemorrhage or septic shock. If this is indeed the case, the rapid loss of muscle mass in ICU patients may more closely reflect the evolutionary origins of hypoxia-induced cachexia and its possible benefits, than the high altitude condition.

## Conclusions and outlook

Sustained exposure to cellular hypoxia, whether in the context of ascent to high altitude or as a consequence of critical illness, threatens cell function and survival. Established features of the hypoxia response include measures to maintain blood oxygen content (e.g. via erythropoiesis) and reduce tissue oxygen demand (e.g. by inhibition of mitochondrial oxidative phosphorylation and possibly by a loss of mitochondrial density).

**Figure 2:**
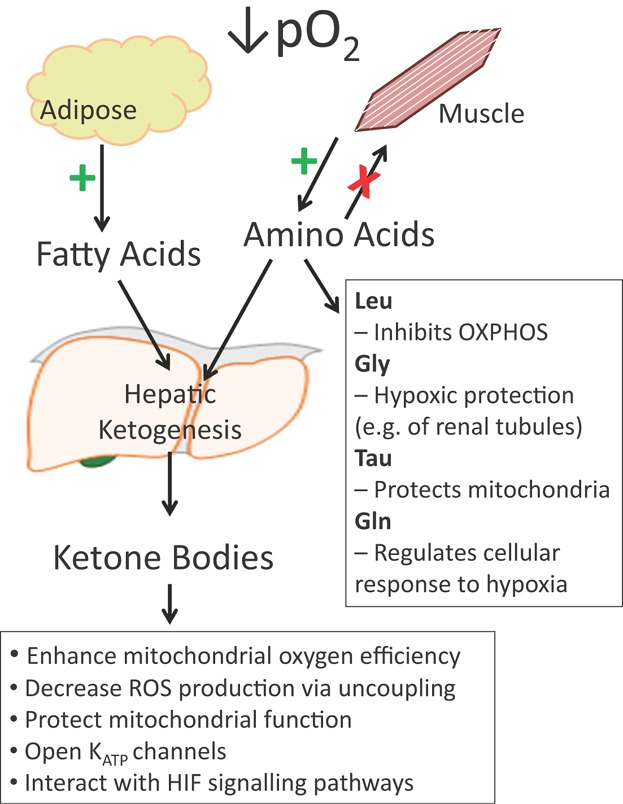
Sustained exposure to hypobaric hypoxia results in cachexia. Fatty acids are released from adipose tissue by lipolysis, whilst muscle breakdown releases amino acids. Fatty acids and some amino acids are converted into ketone bodies by the liver. Ketone bodies and amino acids act as metabolic substrates, but also as metabolic modulators, eliciting protective effects on cells via a myriad of general or tissue-specific mechanisms. Thus, we propose that the hypoxia-induced cachexia at high altitude is protective. Leu, leucine; Gly, glycine; Tau, taurine; Gln, glutamine; OXPHOS, oxidative phosphorylation; ROS, reactive oxygen species; K_ATP_ channels, ATP-activated potassium channels.

We now propose that the catabolic response to sustained hypobaric hypoxia not only contributes to reduced oxygen demand (e.g. by reducing those costs associated with protein synthesis), but also acts to further modulate metabolism and to augment cellular hypoxic protection (Fig. 2). Specifically, we postulate that ketone bodies are synthesized as a result of lipolysis and amino acids released by muscle breakdown; that hypoxia augments both ketone synthesis and amino acid availability; that increased amino acid flux can augment ketogenesis; and that both ketones and amino acids act not only as metabolic fuels, but also as metabolic modulators, offering generalized or tissue-specific hypoxic protection. Ketones decrease the O_2_ cost of ATP synthesis, lessen cerebral ROS production and open K_ATP_ channels, whilst interacting with hypoxia signalling pathways. Meanwhile, certain amino acids protect mitochondria (e.g. taurine) and cellular function in hypoxia (e.g. glycine), whilst glutamine further modulates the cellular response to hypoxia. We propose that such responses may be common to many illnesses at sea level, in which strategies to augment ketone and amino acid delivery might offer therapeutic advantage.

The concepts presented here could be verified using metabolic flux studies in subjects at altitude (real or simulated), and the therapeutic impacts of combined ketone/amino acid delivery assessed in disease states such as critical illness.

## Abbreviations

AcAc: acetoacetate
AMPK: AMP-activated protein kinase
AMS: acute mountain sickness
ARNT: aryl hydrocarbon receptor nuclear translocator
CCK: cholecystokinin
COPD: chronic obstructive pulmonary disease
HIF: hypoxia-inducible factor
ICU: intensive care unit
**K**_**ATP**_**channel**: ATP-sensitive potassium channel
mTOR: mammalian target-of-rapamycin
mTORC1: mTOR complex I
β-OHB: β-hydroxybutyrate
PPARα: peroxisome proliferator-activated receptor α
ROS: reactive oxygen species
RQ: respiratory quotient
SUR: sulfonylurea receptor
UCP: uncoupling protein
